# Medicines support and social prescribing to address patient priorities in multimorbidity (MIDAS): protocol for a definitive, multi-arm, cluster randomised, controlled trial in Irish general practice

**DOI:** 10.1136/bmjopen-2025-101315

**Published:** 2025-06-20

**Authors:** Farah Tahsin, Paul Doody, Barbara Clyne, Bridget Kiely, Frank Moriarty, Paddy Gillespie, Eanna Kenny, Fiona Boland, Molly Byrne, Laura O’Connor, Andrew W Murphy, Susan M Smith

**Affiliations:** 1 Discpline of Public Health and Primary Care, Trinity College Dublin, Dublin, Ireland; 2 Department of Public Health and Epidemiology, Royal College of Surgeons in Ireland, University of Medicine and Health Sciences, Dublin, Ireland; 3 Department of General Practice, Royal College of Surgeons in Ireland, University of Medicine and Health Sciences, Dublin, Ireland; 4 School of Pharmacy and Biomolecular Sciences, Royal College of Surgeons in Ireland, University of Medicine and Health Sciences, Dublin, Ireland; 5 Health Economics & Policy Analysis Centre (HEPAC) at the J.E. Cairnes School of Business & Economics, University of Galway, Galway, Ireland; 6 Data Science Centre, Royal College of Surgeons in Ireland, University of Medicine and Health Sciences, Dublin, Ireland; 7 School of Psychology, University of Galway, Galway, Ireland; 8 HRB Primary Care CTNI, Department of General Practice, University of Galway, Galway, Ireland

**Keywords:** Multimorbidity, Primary Care, Polypharmacy

## Abstract

**Background:**

There is increasing awareness of the impact of living with multiple long-term conditions (referred to as multimorbidity) on patients and health systems. Managing multimorbidity remains a challenge for primary care providers; necessitating tailored interventions that are both clinically and cost effective. In the Irish health system, two pilot trials have demonstrated promising results for patients living with multimorbidity. The first, MultimorbiditY COllaborative Medication Review And DEcision making (*MyComrade*), involved pharmacists supporting the management of polypharmacy, and the second, Link MultiMorbidity (*LinkMM*), involved link workers delivering social prescribing. This definitive trial aims to evaluate the clinical and cost effectiveness of both these interventions, as well as conduct a process evaluation.

**Methods:**

This is a pragmatic, multi-arm, definitive, cluster randomised controlled trial in Irish general practices. The trial will include three arms: (1) MyComrade; (2) LinkMM and (3) usual care, acting as an efficient shared control arm for both interventions. For this trial, 672 patients will be recruited from 48 general practices. The eligibility criteria for the patients will be: (1) over 18 years of age; (2) has two or more chronic conditions; (3) taking 10 or more regular medicines and (4) attending their general practice team for chronic disease management. Outcome data will be collected for all participants, across all trial arms at baseline and 6 months. Primary outcomes include the number of medicines (reflecting the MyComrade intervention) and patient capability (reflecting the LinkMM intervention). Secondary outcomes include proportions and types of potentially inappropriate medications, patient experience of care, patient activation, self-rated health, health-related quality of life, mortality and healthcare utilisation. Quantitative and qualitative data will be collected to inform the process evaluation. Additionally, an economic evaluation will be conducted to evaluate the cost-effectiveness of both interventions compared with the control arm.

**Ethics and dissemination:**

The trial protocol was approved by the Irish College of General Practice (ICGP) Ethical Review Board (ref: ICGP_Rec_2023_016). A formal knowledge dissemination plan has been developed for the trial, which includes peer-reviewed publications, conference presentations and reports to healthcare professionals, commissioners and policymakers.

**Trial registration number:**

ISRCTN11585238.

**Strengths and limitations of the study**

- The Medicines Support and Social Prescribing to Address Patient Priorities in Multimorbidity trial was co-designed, conceptualised and informed by patients living with multimorbidity, which ensures the appropriateness of the trial.
- The multi-arm trial design allows simultaneous assessment of two promising interventions against a single control arm.
- Due to funding limitations and to avoid contamination of the practitioners involved, a combined intervention arm (both LinkMM and MyComrade) would not be delivered in any practices.

## Introduction

 Multimorbidity is the co-occurrence of at least two chronic conditions in an individual. It is associated with poorer health outcomes, higher health service utilisation and is a major challenge to health systems.^1^ There is a clear need for trials of interventions that address the challenges faced by both practitioners and patients in managing multimorbidity.^2^ Qualitative work with patients and practitioners has identified two key areas that interventions could potentially target, namely, addressing polypharmacy and improving patient capacity through non-clinical resources.^3 4^ International clinical guidelines also emphasise the importance of individualised interventions to address the unmet needs of patients living with multimorbidity.^1 5^ However, there is limited evidence on the type of interventions that could better support this patient population.^6^

Evidence suggests that polypharmacy remains a challenge for patients living with multimorbidity.^7^ In response to this challenge, the UK’s National Institute for Health and Care Excellence recommends medicines management interventions addressing the number and appropriateness of medicines be targeted at those with more significant polypharmacy.^1^ This recommendation aligns with more recent evidence from a systematic review,^6^ a large trial on polypharmacy in patients with multimorbidity (Supporting Prescribing in Older Adults with Multimorbidity in Irish Primary Care (SPPiRE)),^8^ and the MultimorbiditY COllaborative Medication Review And DEcision making (MyComrade) pilot randomised controlled trial (RCT).^9^ A previous systematic review identified that integrating practice-based pharmacists reduces potentially inappropriate prescribing (PIP) and the number of medications.^10^ The MyComrade pilot RCT targeted collaborative medication reviews in general practice involving pharmacists and patients taking 10 or more regular medicines and confirmed that practice staff and patients found the intervention acceptable and reported strong fidelity to the intervention components.^9^

Another challenge patients living with multimorbidity experience is a reduced capacity to manage their conditions, which in turn negatively impacts their well-being.^11^ To address this challenge, an exploratory RCT, the link workers in Link MultiMorbidity (LinkMM) study,^12^ evaluated an intervention designed to identify and address priorities for patients living with multimorbidity through supporting connections with non-medical community resources that can enhance health behaviours, activities and well-being. This pilot RCT indicated that practice-based link workers are feasible and acceptable to patients and providers.^13^ Before conducting this trial, a systematic review was conducted of link-worker interventions, indicating that embedding link workers delivering social prescribing into chronic disease management can significantly reduce hospitalisations.^14^

In both the MyComrade and LinkMM pilots, the promise of the potential impact on key patient outcomes was established.^9 13^ However, a definitive cluster RCT is needed to explore the clinical effectiveness and cost-effectiveness of these two promising interventions. Hence, the aim of the Medicines Support and Social Prescribing to Address Patient Priorities in Multimorbidity (MIDAS) cluster RCT will be to evaluate the clinical effectiveness and cost-effectiveness of these two interventions in Irish general practice. Another aim of the trial is to evaluate the implementation process of this trial through a process evaluation.

## Methods

### Study design

**Figure 1:**
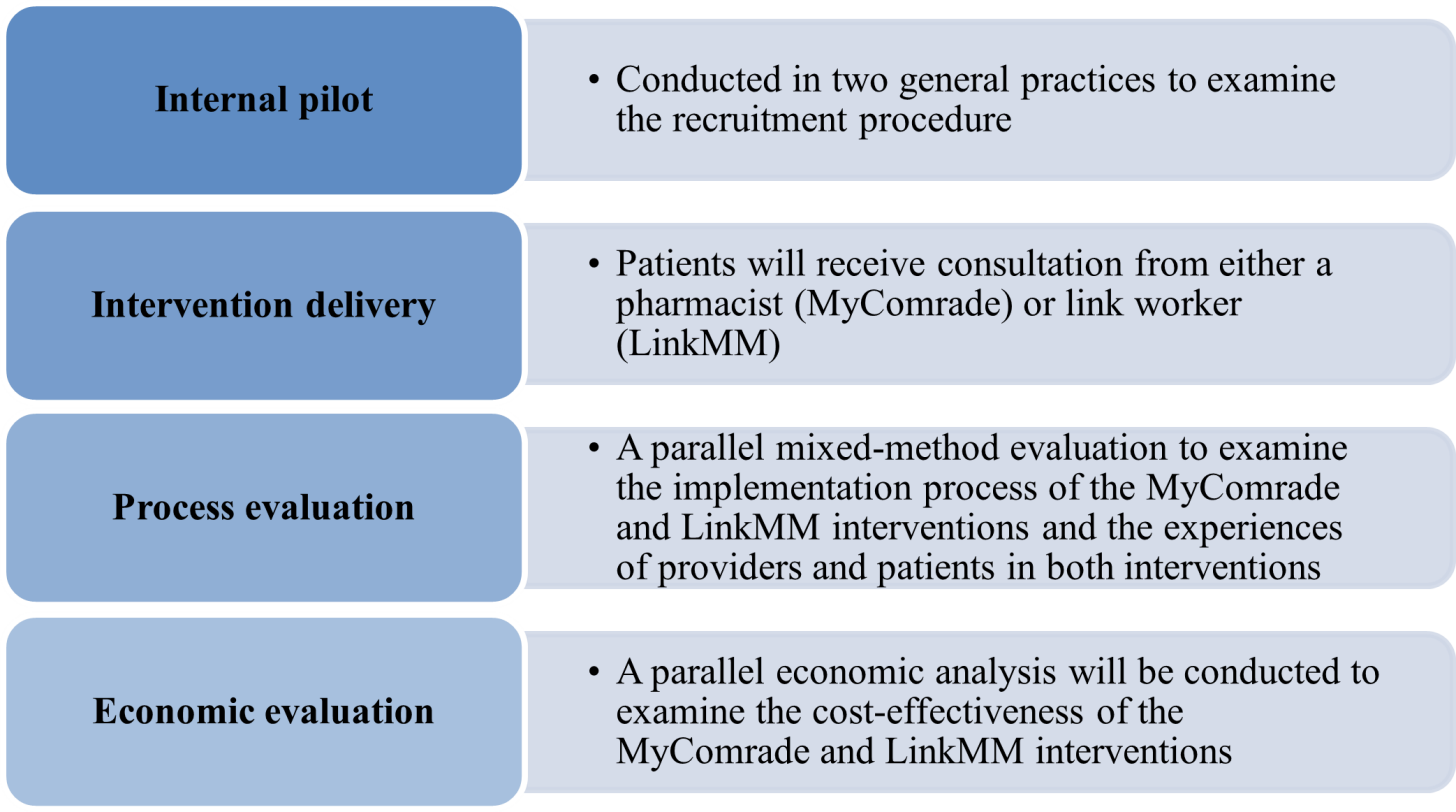
Overview of the main study components.

MIDAS is a pragmatic multi-arm definitive cluster RCT which also includes a process and economic evaluation (figure 1). This trial will be conducted in Irish general practices.

Ireland has a two-tiered healthcare system comprising public and private tiers that provide a wide range of hospital and general practice-based services.^15^ General practice services are provided without charge to approximately 40% of the population (children aged under 8 years, adults aged over 70 years and others based on low-income thresholds) with the remainder paying a cost per consultation of approximately €60.

MIDAS trial will include general practices in Ireland that are enrolled in the national Chronic Disease Management (CDM) programme.^15^ The CDM programme resources general practitioner (GP) teams to conduct bi-annual structured reviews for patients with one of the following chronic conditions: type 2 diabetes, asthma, chronic obstructive pulmonary disease (COPD) or cardiovascular disease (including heart failure, coronary artery disease, stroke and atrial fibrillation) and who are eligible for free GP care.

The first review occurs with a general practice nurse (GPN) who identifies risk factors, undertakes relevant blood tests, explores patient priorities to identify goals, and in doing so develops an agreed care plan with the patient. Afterwards, a GP reviews the patient with a focus on education and medicine management.^15^ The GPN review and subsequent GP review both facilitate the means for integrating MIDAS interventions into the CDM programme. Because of the structured nature of CDM, this programme provides an ideal recruitment setting for the MIDAS trial.

For the LinkMM intervention arm, the GPN can incorporate elements of LinkMM into their CDM reviews, by enhancing standard care through referral to a practice-based link worker skilled in social prescribing. Similarly, for the MyComrade intervention arm, the GP can integrate components of MyComrade, improving usual care with the help of a practice-based pharmacist skilled in medication reviews. This embedded intervention delivery approach underscores the pragmatic nature of the study.^16^

The MIDAS trial will take place from April 2024 to January 2027. The study protocol was prospectively registered on the International Standard Randomised Controlled Trial Number registry. This study protocol has been reported in accordance with the Standard Protocol Items: Recommendations for Interventional Trials (SPIRIT) Statement (online supplemental file 1).

### Internal pilot

While both interventions have been developed with feasibility testing across 16 practices (10 practices for MyComrade,^9^ 6 practices for LinkMM^13^), an additional internal pilot of the specific MIDAS trial processes was conducted. The internal pilot aimed to test the MIDAS trial processes including patient recruitment, study materials and data collection. The internal pilot was conducted in two general practices, one urban (Dublin) and one rural (Galway). The main findings of the internal pilot were as follows: (1) a recruitment training video created by the trial team will be beneficial for carrying out the recruitment process and (2) the number of eligible patients will vary across practices. Additionally, based on feedback from GPNs at each practice, a recruitment procedure was developed for the trial. These findings have been incorporated into the study protocol. The recruitment guideline for each practice can be found in online supplemental file 2.

### Sample and recruitment

#### Recruitment strategy

**Figure 2:**
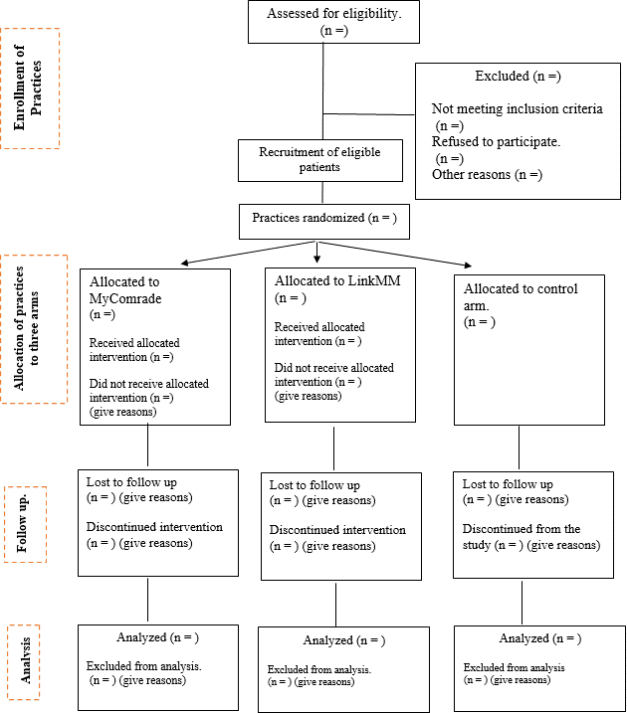
CONSORT flow chart for MIDAS cluster RCT. CONSORT, Consolidated Standards of Reporting Trials; MIDAS, Medicines Support and Social Prescribing to Address Patient Priorities in Multimorbidity; RCT, randomised controlled trial.

A two-stage sampling strategy will be implemented, first recruiting general practices (clusters), followed by recruiting eligible patients within recruited practices. Figure 2 shows the recruitment flow for the study.

#### Practice recruitment

Practice clusters will be recruited from general practices in Ireland that meet the eligibility criteria. The following are the eligibility criteria.

##### Inclusion criteria

1. General practices with at least 250 patients, participating in the CDM programme. This approach will ensure feasible recruitment for the trial.

##### Exclusion criteria

1. General practices that currently have a pharmacist or link worker working within their practice.
2. General practices whose geographic location makes intervention delivery impossible, for example, a single interested practice in a remote rural or semi-rural location, making travel for a pharmacist or link worker to deliver interventions too difficult.

### Patient recruitment

Following the recruitment of general practices, study participants will be recruited.

#### Inclusion criteria

The inclusion criteria for the patients are:

1. Patients aged ≥18 years attending their general practice for a CDM review. The CDM programme is available for patients who have one of the following conditions: type 2 diabetes, asthma, COPD and cardiovascular disease (heart failure, angina, stroke, irregular heartbeat).
2. Patients who have multimorbidity (defined as having two or more chronic conditions). The definition of chronic condition will be based on practice judgement.
3. Patients who are taking 10 or more regular medicines based on repeat prescriptions on electronic patient records.

#### Exclusion criteria

1. Patients who are unable to provide informed consent based on language or serious cognitive impairment (based on known clinical diagnosis).
2. Patients who have limited life expectancy (less than the intervention duration and follow-up period).

The broad inclusion criteria facilitate feasible recruitment as 60% of patients over 50 years of age attending GPs in Ireland have multimorbidity^17^ and 29% of older adults are taking 10 or more regular medicines.^18^

### Practice recruitment procedure

Practice recruitment will occur through the Health Research Board (HRB) Primary Care Clinical Trials Network Ireland (PCCTNI). The PCCTNI team will circulate trial-related information and seek expressions of interest from GPs across Ireland. This recruitment procedure was developed based on the lessons learnt from the previous SPPiRE trial.^8^

### Patient recruitment procedure

**Figure 3:**
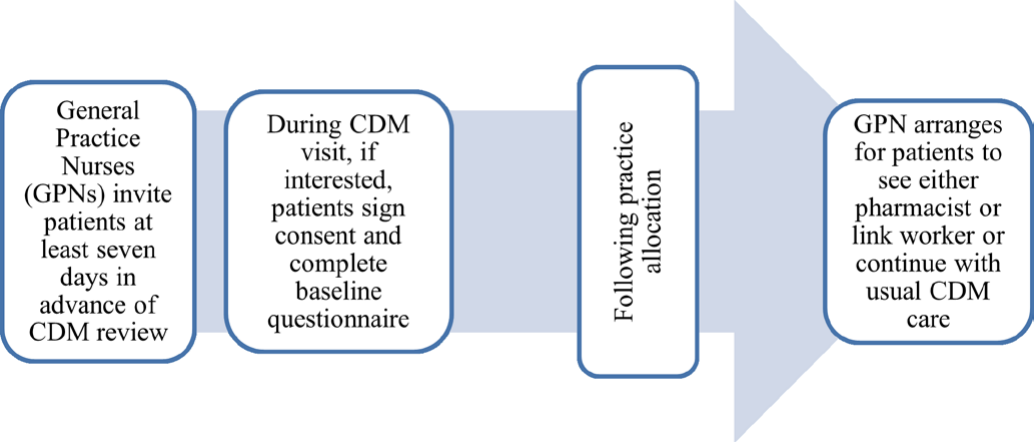
Patient recruitment procedure. CDM, Chronic Disease Management programme.

All patients attending a participating practice as part of the CDM programme will be screened prospectively by the practice team. Eligible patients will be invited to participate by the practice team. When a patient consents to participate, baseline data will be collected prior to practice randomisation, to minimise bias that could arise if participants were aware of the allocation status of their practice. Figure 3 visualises the patient recruitment procedure for the MIDAS trial.

### Practice allocation and randomisation and blinding

After collecting baseline data, a blinded trial statistician (FB) will allocate each practice to a study arm using a minimisation technique.^19^ The allocation will consider variables such as practice size (measured by the number of GP sessions per week: 0–14, 14–28 or 28 or more, with 10 sessions per week representing one full-time equivalent GP) and location (urban, rural or mixed), as successfully done in the previous SPPiRE study.^8^ The randomisation will be performed using Stata. The statistician will remain blinded to the study arm during data analysis.

### Interventions

Intervention delivery will commence in each interventional practice following practice allocation and will continue over the following 6 months as needed for each patient. Patients can withdraw from the intervention at any time. This aligns with how each intervention would be delivered in routine care.

### Description of the interventions

#### Intervention arm 1 (MyComrade)

The main aim of the MyComrade intervention is to support GPs to conduct medication reviews for patients with multimorbidity to optimise the medication regimen and minimise PIP. As part of the intervention, a general practice-based pharmacist (GPP) will be based in the practice for a session a week and will review the recruited patients’ charts. The GPP will engage with the patient, drawing on the 7-steps medication review process.^20^ The seven steps medication review process can be found in table 1.

**Table 1:** Seven steps medication review process^20^

Domain	Steps
Aim	Step 1. What matters to the patient?
Need	Step 2: Identify essential drug therapy
	Step 3: Does the patient take unnecessary drug therapy?
Effectiveness	Step 4: Are therapeutic objectives being achieved?
Safety	Step 5: Does the patient have Adverse Drug Reactions (ADR)/Side Effects or is at risk of ADRs/Side Effects?
Efficiency	Step 6: Is drug therapy cost-effective?
Patient-centredness	Step 7: Is the patient willing and able to take drug therapy as intended?

After conducting the review process, the GPP and GP will review medicines, identify any issues and agree on a plan for medication management. The GP will consult with the patient to implement an agreed-upon medicine plan. Afterwards, the GPP will follow-up with patients as needed.

#### Intervention arm 2 (LinkMM)

The LinkMM intervention is based on the community link practitioners interventions developed by Deep End GPs (GPs serving the most deprived areas of Scotland), which draws on the model of community-oriented primary care.^21^ Link workers are non-health or social care professionals, with skills in listening and motivational interviewing, who support people to develop and implement individual health and well-being plans, often through connection with community resources.^14^ They will be based on general practices for two sessions a week over the 6-month intervention period. Participating patients will meet the link worker in the practice. The link worker and the patient will explore the patient’s priorities and develop a plan to address them, including supported referrals to community resources. The link worker will follow-up as required over the 6-month period to monitor progress and provide coaching and emotional support to achieve the plan. Based on findings from previous systematic reviews and trials,^12 13^ we anticipate that link workers will have at least three meetings per patient but the intensity of the support offered will be tailored to the individual needs of the patient. Four types of support will be provided as follows:

1. Instrumental: doing things for patients.
2. Informational: passing on information.
3. Emotional: listening and supporting.
4. Appraisal: helping people to assess situations and make plans.

### Comparison 

The control arm will receive usual GP CDM care, with no additional support for medicines reviews or addressing patient priorities. A typical GP CDM review involves the following two components: (1) initial consultation with a GPN for a blood test, blood pressure check, weight and height check, and, if necessary, other tests and (2) review with a GP to discuss management of chronic conditions. The primary comparisons will be intervention 1 versus control and intervention 2 versus control.

### Outcomes

The primary and secondary outcomes for the MIDAS trial were selected based on the core outcome set for multimorbidity^22^ and the previous pilot RCTs.^9 13^ The primary outcomes, collected from all participants, are total number of medications per patient (addressing the MyComrade intervention) and patient capability, measured using ICEpop CAPability measure for Adults (ICECAP-A) questionnaire (addressing the LinkMM intervention).^23^ ICECAP-A is a self-reported questionnaire used to measure patients’ quality of life and well-being.^23^ The full list of outcomes is listed in online supplemental file 3. All outcomes are being collected for all participants irrespective of the trial arm at baseline and at 6 months.

### Sample size calculation

The overall sample size was calculated based on the two primary outcomes: number of medications (MyComrade) and patient capability (LinkMM). Based on these estimates, we will aim to recruit 48 GP practices and 672 patients across the three arms of the trial (approximately 16 practices and 224 patients per arm). Detailed calculations for each primary outcome can be found below.

#### Number of medications

The sample size calculation is based on data from the pilot study, supplemented by data from the similar SPPiRE study.^7^ The calculation is based on an absolute mean difference at follow-up in the number of medications between Intervention 1 (MyComrade) and the control arm of the study of 0.8. In the SPPiRE trial, a reduction of 0.85 was seen, and as highlighted by McCarthy *et al*,^8^ although this effect size could be considered small at the individual level, if implemented at scale, it would have a significant impact at a population level. Based on the findings in this study, specifically an SD of the difference in the number of medications of 1.29 and an intraclass correlation coefficient (ICC) of 0.049, and furthermore adjusting for two primary outcomes, approximately 120 patients per arm from eight practices (average 13 patients per practice) would be required to detect this difference with 90% power. As with most trials, there is a potential for unequal cluster (practice) sizes. If the coefficient of variation (CV) of cluster size is <0.23, the effect of adjustment for variable cluster size on sample size is negligible.^18^ In this study, allowing for a minimum practice size of 11 and a maximum of 15 results in an approximate cluster size CV of 1.0. Adjusting the sample size to account for this results in 11 practices and approximately 143 patients per arm of the trial. Allowing conservatively for a dropout rate of 10% (as compared with the 1% reported in the pilot), a definitive trial would require 13 practices and approximately 182 patients per arm.

#### Patient capability (ICECAP-A)

The sample size calculation is based on detecting a 15% improvement in ICECAP-A. Using estimates from LinkMM,^12^ mean (SD) ICECAP-A at baseline was 0.72 (0.20), thus a 15% improvement in ICECAP-A is 0.828. Using an ICC of 0.049, adjusting for multiple primary outcomes, an average cluster size of 13, minimum cluster size of 11, maximum of 15 (estimating a cluster size CV of approximately 1.0), approximately 14 practices and 182 patients per arm would be required to detect this difference with 90% power. Allowing for a conservative 10% loss to follow-up results in 16 practices and approximately 224 patients per arm.

### Data collection

To address the objectives of the trial, quantitative and qualitative data will be collected and analysed. Practice profile data will be collected at baseline. All patient outcome data will be collected at baseline and 6-month follow-up for all trial arms. Once patients have consented to participate, they will complete baseline questionnaires. Participants can do this with practice nurse support. However, if patients prefer to keep their response private from the GP team, they can complete the questionnaire independently and submit it to the practice nurse in a sealed envelope.

In addition to patient data, the practice nurse will collect baseline data on current medications and healthcare utilisation over the past 6 months from GP records using a paper template designed by the trial team. The practice nurse will then send all the data collection forms to the trial team in pre-paid envelopes by post. On receiving the data collection forms, a research assistant will input the data into a password-protected Microsoft Excel file to ensure quality and security.

In terms of follow-up data collection, at 6 months, following allocation, patient and practice follow-up data will be collected. Patient-reported outcome measures will be collected with the support of the practice nurse as at baseline. Follow-up data on medicines and healthcare utilisation will be collected by practice nurses from the GP records. To determine medicines outcomes, prescriptions at baseline and 6 months follow-up will be printed off from the patient record and returned with the follow-up data to the research team. A pharmacist will determine medicine outcomes by analysing the prescriptions collected at baseline and follow-up. The pharmacist will be blinded to the patient allocation to limit observer bias.

At follow-up, we will also collect an interim 3-month prescription for all participants across all study arms to examine any changes during the study period. Collection of prescriptions at three intervals is based on our experience during the SPPiRE trial which suggested considerable fluctuations in prescription for patients with significant polypharmacy. Link workers will also collect data on goal setting and community resource referrals as they deliver the intervention. This data will only be collected within the LinkMM intervention arm and will facilitate evaluations of LinkMM intervention fidelity. Table 2 shows the schedule of enrolment, interventions and assessments, according to the SPIRIT guideline.

**Table 2:** Schedule of enrolment, interventions and assessments

Timepoint	Study period		
	Enrolment	Intervention	Post-intervention
	T_0 (baseline)_	0	T_1 (6 months)_
Enrolment in the study			
Eligibility	X		
Informed consent	X		
Interventions			
MyComrade or LinkMM or control		X	
Assessments			
Baseline variables	X		
Primary outcomes	X		X
Secondary outcomes	X		X

LinkMM, Link MultiMorbidity; MyComrade, MultimorbiditY COllaborative Medication Review And DEcision making.

### Data management

Standardised data collection sheets/processes will be used, along with data consistency checks. At data entry, standardised spreadsheets/databases will be used, which include appropriate validation rules and input restrictions. Data checking will be implemented by the research team, incorporating independent double checks as appropriate and extreme value/outlier/range checks. Once data entry is complete, master data files containing the raw data will be retained with restricted access.

### Data analysis

#### Demographic and baseline characteristics

A Consolidated Standards of Reporting Trials flow diagram will be presented, and appropriate descriptive statistics will be used to describe recruited patients and practices and to investigate comparability of the trial arms at baseline. A table will be presented showing baseline demographic and clinical characteristics for each group separately and for all patients together. For categorical measures, the number of patients and percentage will be presented, and for continuous scales, the mean and SD will be presented. For continuous scales which show evidence of skewness, a median and IQR may also be presented, or substituted for the mean and SD.

### Primary outcomes

There are two primary comparisons in this study, intervention 1 (MyComrade) versus control and intervention 2 (LinkMM) versus control. For these comparisons, the primary analyses will involve intention-to-treat comparisons between the intervention arms and the control. Appropriate random effects regression models (linear, Poisson or logistic) including a random practice effect and stratification variables (study hub, practice size and location) will be used. Results will be presented as point estimates (ORs, difference in means or incident rate ratios) with 95% CIs and p values. Tukey’s procedure will be used to adjust for multiple comparisons.

### Secondary outcomes

For all secondary outcomes, similar analyses as for primary outcomes will be conducted, without any additional adjustment for multiple comparisons between groups. Appropriate random effects regression models (linear, Poisson or logistic) including a random practice effect and stratification variables (study hub, practice size and location) will be used. Results will be presented as point estimates (ORs, difference in means or incident rate ratios) with 95% CIs and p values.

#### Subgroup analysis

For the primary outcome measures, planned subgroup analyses will be conducted using appropriate interactions to evaluate the potential effects of gender, age (<65 vs ≥65 years, which is the usual retirement age in Ireland), socioeconomic status and multimorbidity (>2 vs >3 conditions).

### Process evaluation

A convergent parallel mixed-methods process evaluation will also be undertaken to evaluate the implementation aspect of the trial.^24^ The evaluation will be conducted in accordance with the Medical Research Council guidance for process evaluations of complex interventions.^25^

The aim of the MIDAS process evaluation is to explore the implementation of the MyComrade and LinkMM interventions and the experiences of practitioners and patients involved in both interventions. Specific objectives of this evaluation include:

1. To examine the implementation of the intervention in terms of
  - Reach.
  - Fidelity and dose (ie, delivery of the intervention as intended) and the influences of context on fidelity.
2. To examine intervention receipt and engagement by patients, and contextual factors influencing this.
3. To examine intervention delivery from the perspective of intervention personnel.
4. To explore perceived effectiveness and acceptability of the interventions.
5. To identify unintended consequences of trial.
6. To explore care as usual (control practices).

### Population and data collection

To address the objectives, quantitative and qualitative data sources will be utilised. Quantitative data on reach and fidelity of delivery and receipt of core intervention components and the targeted mechanisms of change of the intervention will be collected during the cluster RCT via patient questionnaires and trial outcome data collection as listed in table 2.

Data on intervention receipt and engagement will also be collected during semistructured interviews, which will be conducted with a purposefully sampled diverse range of participants seeking variation in age, gender, educational attainment, ethnicity and level of adherence to the interventions to broadly and comprehensively understand perceptions, experiences and influences with the intervention, attending to immediate and wider sociocultural and socioeconomic influences. Intervention personnel (pharmacists and link workers) will also keep diaries of their activities, which will provide further detailed information on intervention delivery in terms of the number of meetings, meeting duration and outputs. We will also conduct qualitative interviews with GPs, practice nurses and patients in a smaller selection of control practices on study completion (to avoid contamination of control practices during the intervention period), which will give us information on usual care and developments that have occurred in routine care over the lifespan of the trial to inform our understanding of intervention effects within and across participating practices. Each participant participating in the interviews will be provided with an information leaflet and consent forms prior to the interviews.

Qualitative interviews will be conducted with intervention practitioners (GPs, practice nurses, pharmacists and link workers) and patients following the cluster RCT to explore the fidelity of intervention delivery, including consideration of adherence to and receipt of the MIDAS interventions, and the influences of context on implementation during the trial from multiple perspectives. These interviews will also explore the potential to deliver both interventions in the same practice. The process evaluation will therefore inform the explanation of intervention effects in the cluster RCT, allow us to refine the intervention components and enable us to understand implementation and change processes and the contextual and cross-cultural factors influencing system-wide implementation of the MyComrade and LinkMM interventions, including patient and practitioner views on the potential to combine both interventions in the same practice.

Topic guides will be developed for each participant group and will address aspects of intervention delivery and perceptions of the participants overall. All interviews will be conducted remotely via MS Teams/Zoom and audio recorded.

In addition, we will explore the feasibility of collecting data from a subset of intervention personnel over the intervention period using Mobile Instant Messaging Ethnography (MIME).^26^ This method will involve study researchers connecting remotely (via Threema app) over a pre-specified time period with intervention personnel, prompting them to share their reflections about intervention delivery—what works and what does not, alongside any contextual factors that may have shaped the intervention delivery. The MIME approach would enable intervention personnel to share their ongoing experience with delivering the intervention in ‘real time’ as opposed to a single once-off interview post intervention delivery. Given the intervention period (6 months), ongoing real-time data collection may provide richer data on contextual factors influencing intervention delivery as well as provide an insight into the lived experience of delivering the intervention from the perspective of personnel embedding into a new role in primary care. MIME, as a remote method, is arguably less intrusive than an ethnographic observation study would be, and allows participants some agency over both when and how much to disclose.^26^ However, this approach has not been used in a process evaluation context previously. Hence, it will be a novel methodological contribution for future trials.

### Data analysis

Quantitative data will be analysed using descriptive statistics.

Semistructured interviews will be transcribed and imported into NVivo for thematic analysis following Braun and Clarke.^27^ If feasible to collect, MIME data will be analysed using the same approach. However, this approach also allows for the construction of composite narratives to present an overview of how the intervention was delivered and experienced. Composite narratives in qualitative research involve the use of data from several interviews to tell a story framed as that of a single individual.^28^

### Economic evaluation

A health economic evaluation will be conducted alongside the cluster RCT to evaluate the cost-effectiveness of MyComrade and LinkMM interventions within the MIDAS trial relative to the control. The evaluation will consist of a cost-utility analysis, with health outcomes assessed using quality adjusted life-years (QALYs), estimated using data collected via the EuroQol 5-Dimensions 5-Levels (EQ-5D-5L) at baseline and follow-up, and combined with the Irish EQ-5D-5L value set for Ireland.^29^ In terms of costing, a healthcare system perspective will be adopted as per the guidelines for the conduct of health technology assessment in Ireland.^30^ In addition, private out-of-pocket expenses will be estimated. Data on utilisation of primary, hospital and community care services and private resources will be collected via questionnaires at baseline and follow-up. An incremental analysis will be conducted to calculate the mean differentials in costs and QALYs, and the incremental cost-effectiveness ratio and net benefit statistics. Parametric and probabilistic analysis, in the form of cost-effectiveness acceptability curves, will be employed to explore uncertainty. Exploratory subgroup analyses will be conducted to assess cost-effectiveness within the pre-specified patient groups. The economic evaluation will be reported using the relevant reporting guidelines based on the Consolidated Health Economic Evaluation Reporting Standards.^31^

### Adverse events

In the event that any Serious Adverse Reactions occur (ie, related to the trial interventions or to procedures mandated by the protocol), we will report it to the trial sponsor committee as soon as the Principal Investigator (PI) or study team member becomes aware of the event. The trial will be audited by the sponsor team. Any changes in the trial protocol will be reported to the sponsor committee as well as ethics committee members.

### Patient and public involvement (PPI)

The design of the study has been extensively supported and informed by the PPI group. More specifically, the PPI members have contributed to the design of both interventions, MyComrade and LinkMM, as well as the choice of study outcomes. The PPI group will be continually involved in the intervention implementation process, knowledge dissemination process and trial reporting.

### Ethics and dissemination

The trial protocol was approved by the Irish College of General Practice (ICGP) Ethical Review Board (reference: ICGP_Rec_2023_016). Informed consent will be obtained from all participants prior to enrolment (Patient Consent form: online supplemental file 4). A formal knowledge dissemination plan has been developed, including peer-reviewed publications, conference presentations and reports to healthcare professionals, commissioners and policymakers. Results will also be shared with participating sites and made available in accessible formats for patients and the public.

## Discussion

Supporting patients with multimorbidity in general practice and primary care settings remains a challenge for healthcare systems across the globe. This pragmatic cluster RCT will evaluate both the clinical effectiveness and cost-effectiveness of two promising interventions. The findings of this trial will be beneficial to understand whether such interventions could be beneficial for patient outcomes as well as reduce healthcare costs. Additionally, the embedded process evaluation will be beneficial to highlight how the interventions were delivered and experienced and whether components of interventions need to be modified or improved to better support patients living with multimorbidity in general practices.

The pragmatic nature of the MIDAS trial will enhance the findings of this trial which are applicable to other settings. For example, the embedded design within the existing national CDM programme provides a unique opportunity and pathway for potential system-wide implementation to support patients with multimorbidity. Additionally, similar CDM programmes exist in many developed countries, and multimorbidity remains a global challenge. Therefore, this trial has the potential for successful interventions to be implemented in other healthcare systems. Moreover, this trial was designed and is being guided by a dedicated PPI group, which will be beneficial to enhance the patient-centredness of both trial design, such as recruitment materials, as well as knowledge dissemination products.

### Trial status

Protocol version number: version 2; Date: 11 May 2024. Practice recruitment completion: 30 April 2025. Patient recruitment completion: 30 June 2025.

## Supplementary material

10.1136/bmjopen-2025-101315 online supplemental file 1

10.1136/bmjopen-2025-101315 online supplemental file 2

10.1136/bmjopen-2025-101315 online supplemental file 3

10.1136/bmjopen-2025-101315 online supplemental file 4
