# Supplementary material for: Medicines support and social prescribing to address patient priorities in multimorbidity (MIDAS): protocol for a definitive, multi-arm, cluster randomised, controlled trial in Irish general practice
Source: BMJ Open. 2025 Jun 20;15(6):e101315. doi: 10.1136/bmjopen-2025-101315 (PMC12182153; doi:10.1136/bmjopen-2025-101315)
Supplement: online supplemental file 2 [file bmjopen-15-6-s002.docx]

| Medicines Support and SocIal Prescribing to aDdress pAtient priorities in multimorbidity (MIDAS):  A cluster randomized trial in Irish general practice |
| --- |

Practices have systems in place to identify CDM patients. These can be adapted to address MIDAS patient recruitment. The following are the key steps:

1. Identify all patients coming in for CDM review at least one week in advance [Record this number on the Patient Recruitment form].
2. Screen these patients for MIDAS eligibility [Record this number on the Patient Recruitment form].
3. Either invite all eligible patients or select a random selection if the practice can not manage higher numbers [please discuss this with the trial manager if needed].
4. Send study information to those being invited.
5. Call those invited to ascertain potential interest.
6. Arrange extended CDM GP Nurse consultations.
7. When potential participants arrive at the GP surgery for CDM appointment, reception can hand them the patient information leaflet, consent, and baseline questionnaire to review.

*Once the patient has received and reviewed these forms:*

1. The patient meets the GP nurse who goes through the Patient Information Leaflet and consent form.
2. Once consent is fully given, the baseline questionnaire is completed either with the nurse or by the participant in the reception area and returned to reception or the nurse.
3. If the patient prefers to take the baseline questionnaire home for completion, this is permissible, but they need to be brought back into the practice.
4. GP Nurse will check completion of questionnaire.
5. GP Nurse will ensure all study documents have been completed with Study ID for patient and will store all documents securely in the practice.

**Two examples of Practice Strategies for Patient Recruitment Process:**

| ***Patient Recruitment: Practice A Example*** |
| --- |

**Step 1: Pre-Consent**

- GP nurse checks who is due for CDM review in the next 2-week block (a week in advance).
- GP nurse creates a list of all those due for CDM in that 2-week period (and records total number of patients due for CDM).
- GP nurse screens this list to check on MIDAS eligibility (and records total number of patients eligible for the MIDAS trial).
- GP nurse sends out MIDAS brief patient invitation letter or text to eligible patients with a reminder for their CDM appointment. This information needs to be received by the patient at least seven days in advance of their appointment.
- Admin/nurse rings patients to see if they are potentially interested so as to allow for a longer CDM appointment time (appointment length depends on CDM complexity but the plan is to allow an additional 10-20 mins for MIDAS activities).

**Step 2: Interested potential MIDAS participants attend the practice for their CDM review**

- On arrival, reception gives the interested patient the full PIL, consent form and questionnaire to review while they wait.
- Patient sees the GP nurse who confirms interest and, if patient agrees, collects consent including giving the PIL and copy of the consent form to the patient for their own records.
- Participants can complete the baseline questionnaire themselves or with support of the GP nurse ***BUT*** the GP nurse is to check the questionnaire is completed fully before the patient leaves.
- If the participant prefers, they can take away the questionnaire. If they do this, it is to be returned to the practice and checked by the GP nurse.

**Step 3: MIDAS admin**

- Assign Study ID to patient based on Study ID on the Consent form and questionnaire for that patient.
- Double check the consent form and questionnaire are fully completed.
- GP nurse to keep the completed patient questionnaire securely in the study folder.
- GP nurse to maintain a list of included patients is to be kept securely in the practice, providing a link to patient contact details for future follow up.

**Step 4: Complete recruitment**

Once all 14 patients are recruited, admin is to return the participant consent forms, participant contact list matched to Study ID, and participant questionnaires to the study team via registered post

| ***Patient Recruitment: Practice B Example*** |
| --- |

**Step 1: Pre-Consent**

- CDM bookings are made six months in advance and two weeks prior patients are contacted by post by a receptionist;
- GP nurse will review these bookings and check MIDAS eligibility (and record numbers of patients eligible on a flow sheet);
- Eligible patients will be sent MIDAS brief patient invitation letter together with their usual practice CDM invitation;
- Two days prior to appointment, the receptionist, as usual, rings each CDM patient to confirm they are coming. For eligible patients, the receptionist will also confirm whether they are, in principle, interested in MIDAS. If they are, she will an allocate extra 10 minutes to this CDM appointment.

**Step 2: Potentially interested MIDAS participants attends the practice for their CDM review**

- On arrival, reception provides interested patients the full PIL, consent form, and questionnaire to review while they wait;
- Patient sees GP nurse who confirms their interest and if the patient agrees, collects signed participant consent including giving the PIL and a copy of the consent form to the patient for their own records;
- Patients can complete the questionnaire themselves or with support of the GP nurse ***BUT*** the GP nurse must check the questionnaire is completed fully before the participant leaves the surgery;
- If patient prefers, they can take the questionnaire away and return it later to the practice and the GP nurse will check it is fully completed.

**Step 3: MIDAS record keeping**

- Assign Study ID to patient, based on practice Study ID on the consent form and baseline questionnaire for that patient;
- Double check the consent form and questionnaire are fully completed;
- GP nurse to keep the completed questionnaire securely in the study folder;
- A list of included patients is to be kept securely in the practice – providing a link to patient contact details for future follow up.

**Step 4: Data sharing**

Once all 14 patients are recruited, the practice is to return the study flowsheet, participant consent forms, participant contact list matched to study ID, and questionnaires to the study team via registered post.
