# Supplementary material for: Medicines support and social prescribing to address patient priorities in multimorbidity (MIDAS): protocol for a definitive, multi-arm, cluster randomised, controlled trial in Irish general practice
Source: BMJ Open. 2025 Jun 20;15(6):e101315. doi: 10.1136/bmjopen-2025-101315 (PMC12182153; doi:10.1136/bmjopen-2025-101315)
Supplement: online supplemental file 3 [file bmjopen-15-6-s003.docx]

*Table 2: Outcomes and data sources*

| **Outcomes measures** | **Description of the variables** | **Data source** |
| --- | --- | --- |
| **Primary outcomes** | | |
| Number of medicines (MyComrade) | Numeric | Patient record |
| Patient capability (LinkMM) | ICECAP-A^a^ (1) | Self-reported questionnaire |
| **Secondary Outcome** | | |
| Quality of life | EQ-5D-5L^b^ (2) | Self-reported questionnaire |
| Mental health outcomes | SWEMWBS^c^ (3) | Self-reported questionnaire |
| Treatment Burden | Treatment burden questionnaire (4) | Self-reported questionnaire |
| Patient experience of care | PACIC^d^ (5) | Self-reported questionnaire |
| Patient activation measure | PAM^e^ (6) | Self-reported questionnaire |
| **Medicines Outcomes** | | |
| Number of repeat medications | Numeric count of medications | Patient records |
| Potentially Inappropriate Prescribing (PIP) and high-risk prescriptions | Numeric count of medications | Patient records (repeat prescriptions identified on review by research pharmacists) |
| **Healthcare utilisation** | | |
| GP use | Number of GP visits (both in-person and virtual) | Patient records |
| Emergency department visits | Number of emergency department visits. Date of visits to allow for analysis of time to first visit if appropriate and time to re-visit in days. | Patient records and self-report in previous 6 months |
| Hospital admissions | Number and rates of admission. Length of stay in days if admitted. Time to first admission and time to re-admission in days. | Patient records and self-report in previous six months |
| GP nurse visits | Number of GP nurse visits | Patient records |
| Other primary care use (Allied Health) | Number of visits to other primary care services and identification of those services | Patient records and self-report |
| Out-patient patient department visits | Number of visits to other primary care services | Patient records and self-report |
| Community support visits | Number of visits to other community social services | Patient records and self-reports |
| **Cost Data** | | |
| Healthcare utilisation | As outlined above | Patient record |
| Patient costs | The cost associated with travel and occupation | Self-report |
| Staff costs | Costs related to the recruitment and training of the pharmacist, training of the GPs and associated staff will be taken into account. | Costs per hour (for GP/nurse/secretary input) and per day for pharmacist sing information about salary HSE pay grades based on for each grade of staff |
| Medication costs | Prices for drugs supplied through the community drug schemes are listed in the reimbursement files of the Primary Care Reimbursement Service (PCRS) | PCRS and patient record |

^a^ ICECAP-A: ICEpop CAPability measure for Adults

^b^ EQ 5D 5L: EuroQol 5-Dimensions 5-Levels

^c^ SWEMWBS: Short Warwick-Edinburgh Mental Well-being Scale

^d^ PACIC:Patient Assessment of Chronic Illness Care

^e^ PAM: Patient Activation Measures

Bibliography:

1. Rencz F, Mitev AZ, Jenei B, Brodszky V. Measurement properties of the ICECAP-A capability well-being instrument among dermatological patients. Qual Life Res. 2022;31(3):903–15.

2. Hobbins A, Barry L, Kelleher D, Shah K, Devlin N, Goni JMR, et al. Utility Values for Health States in Ireland: A Value Set for the EQ-5D-5L. Pharmacoeconomics. 2018;36(11):1345–53.

3. Stewart-Brown S, Platt S, Tennant A, Maheswaran H, Parkinson J, Weich S, et al. The Warwick-Edinburgh Mental Well-being Scale (WEMWBS): a valid and reliable tool for measuring mental well-being in diverse populations and projects. Journal of Epidemiology & Community Health. 2011 Sep 1;65(Suppl 2):A38–9.

4. Duncan P, Murphy M, Man MS, Chaplin K, Gaunt D, Salisbury C. Development and validation of the Multimorbidity Treatment Burden Questionnaire (MTBQ). BMJ Open. 2020 Dec 1;8(4):e019413.

5. Glasgow RE, Wagner EH, Schaefer J, Mahoney LD, Reid RJ, Greene SM. Development and validation of the Patient Assessment of Chronic Illness Care (PACIC). Med Care. 2005 May;43(5):436–44.

6. Patient Activation Measure ® (PAM ® ) 13 License Materials [Internet]. [cited 2020 Jul 28]. Available from: www.insigniahealth.com
