# Supplementary material for: Medicines support and social prescribing to address patient priorities in multimorbidity (MIDAS): protocol for a definitive, multi-arm, cluster randomised, controlled trial in Irish general practice
Source: BMJ Open. 2025 Jun 20;15(6):e101315. doi: 10.1136/bmjopen-2025-101315 (PMC12182153; doi:10.1136/bmjopen-2025-101315)
Supplement: online supplemental file 4 [file bmjopen-15-6-s004.docx]

**Supplementary File 4 (Patient consent form)**

**PATIENT CONSENT FORM**

| **Study title:** Medicines Support and SocIal Prescribing to aDdress pAtient priorities in multimorbidity (MIDAS): A cluster randomized trial in Irish general practice |
| --- |

| I have read and understood the **Information Leaflet** about this research project. The information has been fully explained to me and I have been able to ask questions, all of which have been answered to my satisfaction. | **Tick** |
| --- | --- |
| I understand that I don’t have to take part in this study and that I can opt out at any time. I understand that I don’t have to give a reason for opting out and I understand that opting out won’t affect my future medical care. | **Yes ** |
| I understand that depending on the involvement of my GP practice in the study, a pharmacist or a link worker may invite me to meet with them. I understand that I can opt out of this contact at any stage. | **Yes ** |
| I have been given a copy of the Information Leaflet and this completed consent form for my records. | **Yes ** |
| \| I understand that Trinity College Dublin is the Data controller for this study \|  \| \| --- \| --- \| | **Yes ** |
| **Consent** |  |
| I consent to take part in this research study having been fully informed of the risks, benefits and alternatives. | **Yes **  **No ** |
| I give permission for researchers from Trinity College Dublin to look at my medical records to get information. I have been assured that information about me will be kept private and confidential. | **Yes **  **No ** |
| I consent to information about me (data concerning my health, data collecting during an interview if I take part in one etc.) being used for this research study. | **Yes **  **No ** |
| I consent to be contacted by researchers as part of this research study | **Yes **  **No ** |
| I give permission for the researchers to contact me about taking part in a short interview. I understand that taking part in an interview is optional. | **Yes **  **No ** |

| |

----------------------------------------------------------------------------------------------------------------------------

Patient Name (Block Capitals) | Patient Signature | Date

**To be completed by the Principal Investigator or nominee.**

I, the undersigned, have taken the time to fully explain to the above patient the nature and purpose of this study in a way that they could understand. I have explained the risks involved as well as the possible benefits. I have invited them to ask questions on any aspect of the study that concerned them.

| | |

----------------------------------------------------------------------------------------------------------------------------Name (Block Capitals) | Qualifications | Signature | Date

2 copies to be made: 1 for patient, 1 for PI.
